# Tuberculosis in Healthcare Workers: A Matched Cohort Study in Taiwan

**DOI:** 10.1371/journal.pone.0145047

**Published:** 2015-12-17

**Authors:** Sung-Ching Pan, Yee-Chun Chen, Jann-Yuan Wang, Wang-Huei Sheng, Hsien-Ho Lin, Chi-Tai Fang, Shan-Chwen Chang

**Affiliations:** 1 Institute of Epidemiology and Preventive Medicine, College of Public Health, National Taiwan University, Taipei, Taiwan; 2 Department of Internal Medicine, National Taiwan University Hospital, Taipei, Taiwan; 3 National Taiwan University College of Medicine, Taipei, Taiwan; Johns Hopkins Bloomberg School of Public Health, UNITED STATES

## Abstract

**Background:**

Proportional mortality ratio data indicate that healthcare workers (HCWs) have an elevated tuberculosis (TB) mortality. Whether this is caused by an increased TB incidence, a worse TB treatment outcome, or a combination of effects, remains unclear. To elucidate the hazard components of occupational TB, we assessed TB incidence and TB treatment outcome among HCWs in Taiwan.

**Methods:**

We compared the incidence of active TB among HCWs at a major medical center in Taiwan with that of Taiwan general population in 2004–2012. We also compared the TB treatment outcome of HCWs with that of age/sex-matched non-HCW patients treated at the same hospital, as well as that of nationally registered TB patients.

**Results:**

The standardized TB incidence ratio of the HCWs was 1.9 (95% confidence interval [CI]: 1.2–2.9), compared with the general population. HCWs with pulmonary TB (n = 30) were less likely to have underlying diseases, delay in diagnosis, delay in treatment, or side effects of treatment, compared with age/sex-matched non-HCW TB patients (n = 120) (all Ps<0.05). The TB treatment outcome of HCWs was significantly better than that of non-HCW patients (TB-related mortality: 0.0% vs. 5.8%, P = 0.008, Mantel-Haenszel test). The standardized TB-related mortality rate was 1.08% [95% CI: 0.96% - 1.20%] for all of the nationally registered TB patients in Taiwan.

**Conclusions:**

HCWs are at increased risk of active TB, compared with general population. To mitigate this occupational hazard, more efforts need to be directed towards the prevention of nosocomial TB transmission. Healthy worker effect, more rapid diagnosis, and less delay in treatment contribute to a lower TB-related mortality in HCWs.

## Introduction

Health care workers (HCWs) are at risk of exposure to infectious tuberculosis (TB) patients, with an annual rate of incident latent TB infection ranging from 0% to 11.3% (low-income countries) or from 0.1% to 11.8% (high-income countries) [1]. The risk is correlated with the number of TB cases at health care facilities, and is influenced by the infection control policies [1]. HCWs are also at risk for active TB disease, with a rate 1.8 to 20.0-fold higher than that observed in the general population [1].

Two studies from the United States report a significantly increased proportional mortality ratio (PMR) (ranging from 1.2 to 3.5) attributed to TB, in contrast with other causes of death among HCWs compared with workers in other industries [2, 3]. Nevertheless, a significantly increased PMR should be interpreted with caution because such an increase could be caused by an increase in incidence, a worse treatment outcome, or a combination of effects related to the incidence and prognosis. To elucidate the hazard components of occupational TB among the HCWs, it is necessary to separately determine both the risks and the mortality rate of TB. Surprisingly, there are few data regarding TB treatment outcomes in HCWs [4].

Taiwan is a developed country with a moderate TB burden (an annual incidence of 50–70 per 100,000 population in 2004–2012) [5]. TB is a potential occupational hazard for HCWs in Taiwan [6, 7]. The present study aimed to evaluate both the risks and treatment outcomes of TB among HCWs in Taiwan, using the general population and non-HCW TB patients as the reference groups.

## Methods

### Study Design

This is a retrospective matched cohort study. The study has two parts. First, we compared the incidence of active TB among HCWs in 2004–2012 at Medical Center A with that of Taiwan general population. Second, we compared the outcomes of anti-TB treatment in HCWs with that of non-HCW patients who received anti-TB treatment at the same hospital, matched by age, sex, and the onset year. We also compared the outcomes of anti-TB treatment in HCWs with national TB mortality data.

### Setting

Medical Center A, with a capacity of 2,600 beds, provides both primary and tertiary referral care in Taiwan. The hospital has 5,000–6,000 certified HCWs, including physicians, nurses, therapists, pharmacists, technicians, clerks, and cleaning staff.

### Ethics Statement

The study protocol was reviewed and approved by Research Ethics Committee of National Taiwan University Hospital. The Committee approved the exemption of informed consent. Patient records/information were anonymized prior to analysis.

### Surveillance of TB among HCWs

To facilitate early TB diagnosis, Medical Center A provided (1) annual chest radiography (CXR) for high-risk HCWs since 2002 and for all HCWs (including certified staff, out-sourcing staff and volunteers) since 2006; and (2) contact tracing for TB-exposed HCWs since 1997.

### Diagnostic Procedures for TB

For patients or HCWs with TB-associated symptoms, a sputum acid-fast smear (AFS) and mycobacterial culture were routinely performed. Imaging examinations included CXR or chest computed tomography. For extrapulmonary TB, biopsy was performed for histology examination and mycobacterial culture [8].

### Notification of TB

TB is a notifiable disease in Taiwan. Physicians are required to report all TB cases that are diagnosed at Medical Center A to its Center for Infection Control (CIC), which reports the data to the Taiwan Centers for Diseases Control (CDC). If HCWs are diagnosed with TB at other health care facilities, the local public health station will routinely notify the CIC of the hospital.

### Inclusion Criteria

All of the HCWs at Medical Center A who fulfilled the following two criteria were included as HCW-TB cases: 1) an individual reported to Taiwan CDC as having active TB (including culture-proven and clinically suspected TB); and 2) an individual who had received anti-TB treatment. Clinically suspected cases that had been disproven by subsequent evaluations were excluded.

### Comparison Group for Incidence Study: General Population

We compared the incidence rate of active TB (including pulmonary and extrapulmonary TB) among HCWs at Medical Center A to that of the general population in Taiwan by the indirect standardization method. Data on numbers of certified HCWs are available from the Hospital Personnel Office since 2006. Data on HCWs TB cases occurred in 2004–2012 were obtained from the Hospital CIC. Vital statistic data are publicly available from Ministry of the Interior, Taiwan [9]. Age and sex-specific TB incidence rates of general population are available from Taiwan CDC [5].

### Comparison Group for Outcome Study: Non-HCW Patients

We compared the outcomes of anti-TB treatment in HCWs with that of non-HCW patients at the same hospital. For each HCW case (with pulmonary TB), four non-HCW pulmonary TB patients were randomly selected, matched by diagnosis year, sex and age group (21–30 years, 31–40 years, 41–50 years, 51–60 years, or 61–70 years).

#### Clinical Data

We systematically reviewed medical records to obtain clinical data. The following data were collected: age; sex; underlying diseases; concurrent medications; disease severity (cavitary lesion on CXR or the presence of concurrent extra-pulmonary TB); anti-TB regimen (use of standard isoniazid (INH), rifampin (RIF), ethambutol (EMB), pyrazinamide (PZA) [HERZ] regimen) [8]; whether initial treatment including HERZ was modified due to an adverse event or susceptibility report; treatment duration; and treatment response.

#### Definition of Delay in Diagnosis or Treatment

Patient delay was defined as the duration from symptom onset to the first time that the patient visited the out-patient clinic or emergency department (outside or within Medical Center A). Physician delay was defined as the duration from the first time that the patient visited due to TB-associated symptoms to the date that the diagnostic tests were arranged (including chest X-ray, sputum AFS, *Mycobacterium* culture or biopsy). Treatment delay was defined as the duration from the time that the physician prescribed the diagnostic tests to the time that the anti-TB treatment was actually prescribed. Total delay was defined as the total duration from the time that the patient started to have symptoms to the time when the anti-TB treatment was prescribed [10].

#### Microbiology Data

Culture and susceptibility test results were collected retrospectively through medical record reviews. Routine susceptibility testing is performed according to a standard method [11], in the presence of low and high concentrations of INH (0.2 μg/mL or 1 *μg*/mL), low and high concentrations of EMB (5.0 *μg*/mL or 10.0 *μg*/mL), and RIF. Multidrug-resistant (MDR) TB was defined as TB that was resistant to both INH and RIF [12].

#### Outcome Measures

The primary outcome is TB-related mortality. The death was considered to be TB-related if (1) TB was listed as a cause of death on the death certificate, or (2) death occurred within 30 days after TB diagnosis. The secondary outcomes are frequencies of adverse events during the anti-TB treatment, including blurred vision, skin rash or itching, gastrointestinal upset, malaise, acute kidney injury (defined as an absolute increase in serum creatinine to twice the baseline level or a percentage increase in serum creatinine ≥50%, according to the RIFLE criteria), hyperuricemia (defined as serum uric acid ≥7.5 mg/dL) [13], neutropenia (defined by an absolute neutrophil count [ANC] of less than 500/μL or an ANC of less than 1000/μL with a predicted decline to 500/μL), and hepatitis (defined as alanine aminotransferase level of ≥3-fold of the normal value) [8].

### Comparison Group for Outcome Study: National TB Data

We compared the TB-related mortality rate (including pulmonary and extrapulmonary TB) among the HCWs at Medical Center A to 2004–2012 national TB data in Taiwan. The age- and sex-specific TB mortality rate (mortality cases/TB registered cases) are available from Taiwan CDC [14]. To make the mortality rates comparable, both rates (of HCWs TB and national registered TB patients) were age- and sex-standardized using certified HCWs of Medical Center A in 2012 as the reference population.

### Statistical Analysis

Standardized incidence ratio was used to compare incidences of active TB in HCWs and that of general population. Expected numbers of active TB cases (I*P) are calculated based on the age- and sex-specific TB incidence (I) of general population, multiplied by numbers of age- and sex-specific population at Medical Center A (P), and it was compared with the observed number of incident TB cases among HCWs. Poisson regression was used to evaluate changes in TB incidence among HCWs. For the outcome analysis, differences in TB-related mortality between TB-HCWs and matched non-HCWs TB patients was assessed by Mantel-Haenszel test for matched binomial data. The 95% confidence interval of standardized mortality rate is calculated using established formula [15].

Statistical analyses were performed with STATA 11.0 (College Station, Texas, USA). To compare the characteristics between the matched groups, continuous variables were assessed using the paired t-test for matched data. All of tests were 2-tailed, and *P*-values of less than 0.05 were considered statistically significant.

## Results

### TB Diagnoses in HCWs

From January 1, 2004, to December 31, 2012, a total of 44 HCWs were reported to the CIC of the hospital as active TB cases. Based on clinical and microbiology evaluations, TB was subsequently excluded in three of the 44 HCWs, and these cases were not included in the incidence analysis. Among the remaining 41 TB-HCWs, 26.8% (11/41) were smear positive, 70.7% (29/41) were culture proven, 9.8% (4/41) were pathologically diagnosed, and 24.4% (10/41) were clinically diagnosed. All 41 of these HCWs were native-born Taiwanese. Their occupational categories included 12 physicians, 17 nurses, 6 clerks and 6 other occupational categories (including 3 cleaning staff members and 3 volunteers). Table 1 shows the age and sex distributions of HCWs with active TB. No epidemiological TB disease clustering among the HCWs was observed during the study period.

**Table 1:** The epidemiologic profile of HCWs with active TB during the 2004–2012 period.

Occupation	Physician	Nurses	Clerks	Others*
	(n = 12)	(n = 17)	(n = 6)	(n = 6)
Age	39.8±16.0	34.8±12.5	44.8±7.9	51.0±8.6
Male	8 (66.7%)	0 (0%)	2 (33.3%)	2 (33.3%)
Pulmonary TB	8 (66.7%)	13 (76.5%)	6 (100%)	5 (83.3%)

*The personnel in ‘others’ included 3 cleaning staff members and 3 volunteers.

### TB Incidence

The age and sex stratification among certified HCWs at Medical Center A was available since 2006. After excluding the 10 HCWs who were diagnosed in 2004–2005 and the 9 HCWs who were not certified personnel (i.e. study nurses and outsourced personnel), 22 certified HCWs were diagnosed as having active TB disease in 2006–2012 (Table 2).

**Table 2:** Incidence of TB among HCWs in Medical Center A from 2006 to 2012.

Year	Incident TB cases in certified HCWs at Medical Center A^a^	Number of certified HCWs at Medical Center A	Incidence at Medical Center A (per 100,000 person-year)	SIR^b^ (95% CI)
2004	5	N/A	-	-
2005	5	N/A	-	-
2006	6	4,225	142.0	3.11
2007	2	4,325	46.2	1.13
2008	2	4,851	41.2	1.13
2009	5	5,031	99.4	3.16
2010	3	5,172	58.0	1.92
2011	2	5,449	36.7	1.49
2012	2	5,807	34.4	1.37
Average^c^			63.1	1.93 (1.21–2.92)

^a^Among the 41 TB-HCWs, 5 TB-HCW cases in 2004 and 5 in 2005 were not included because hospital-obtained age and sex information was not available for that period. Another 9 HCW TB cases were further excluded because they were not certified HCWs (2, 1, 1, 2, 1, and 2 cases were excluded in 2007, 2008, 2009, 2010, 2011, and 2012, respectively).

^b^SIR: standardized incidence ratio. The reference is the age/sex-specific TB incidence among Taiwan general population in the same year.

^c^Average TB incidence from 2006–2012

The crude incidence rate among HCWs was 63.1 cases/100,000 population in 2006–2012. Compared with general population in Taiwan in the same period, the standardized incidence ratio is 1.9 (95% CI: 1.2–2.9) (S1 Table), which is equivalent to an adjusted incidence rate of 135.7 cases/100,000 population (95% CI: 20.9–205.8/100,000 population).

Further analysis by year shows a 78% decrease in crude TB incidence among HCWs from 142.0 per 100,000 population (2006) to 34.4 per 100,000 population (2012) (P = 0.10, Poisson regression) (Table 2). Using the TB incidence in general population in the same year as the reference, there is also a 56% decrease in standardized incidence ratio of HCWs from 3.11 (2006) to 1.37 (2012) (Table 2).

### Outcomes: Comparison with Matched Non-HCWs TB Patients

**Fig 1:**
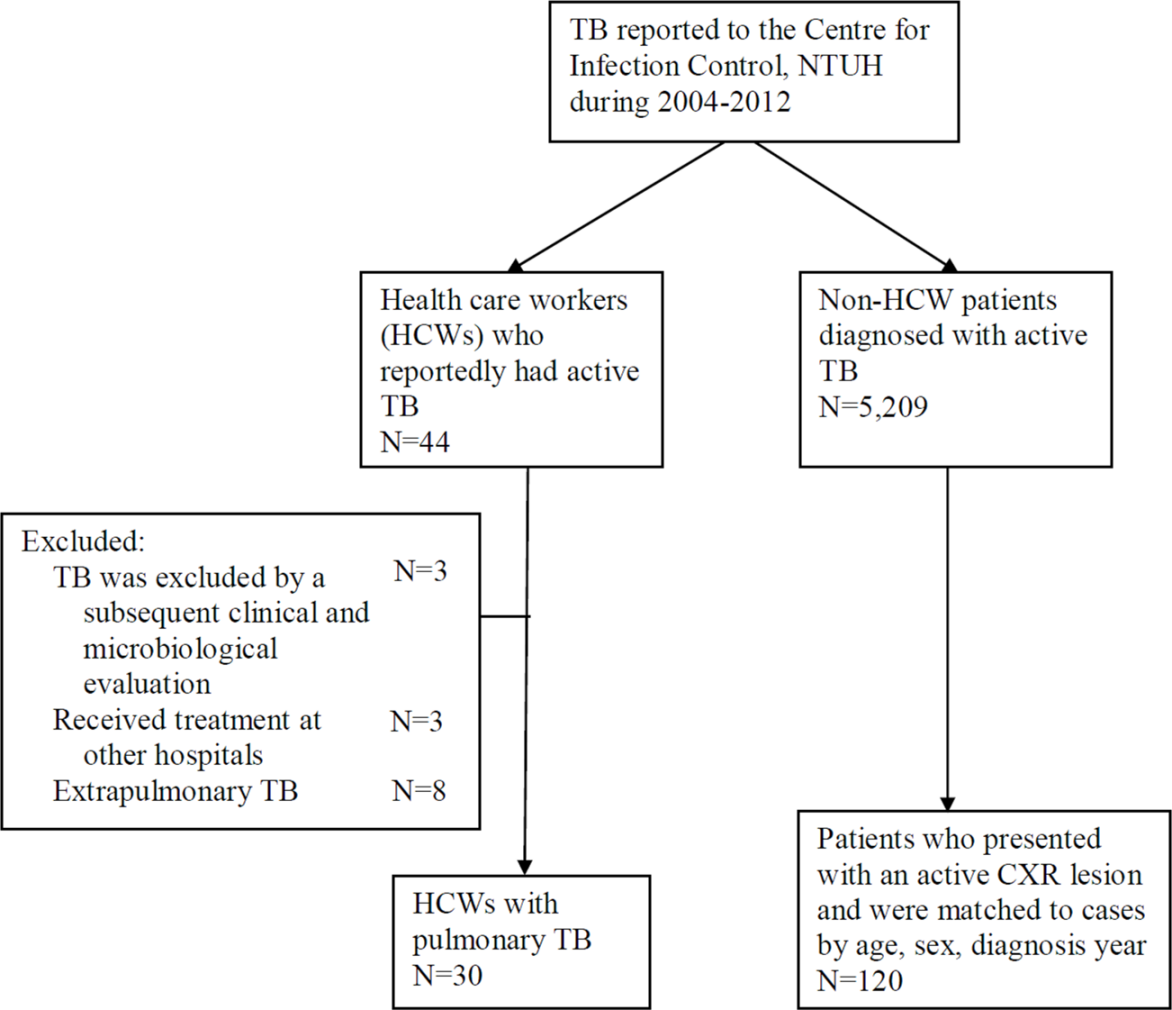
Summary of the recruitment process.

Among the 41 HCWs who had active TB, 3 received treatment at other hospitals, and another 8 had localized extrapulmonary TB diseases (4 with lymphadenopathy and 4 with bone/joint involvement); these 11 HCWs were excluded. In total, 30 HCWs with pulmonary TB were available for outcome analysis, and these subjects were matched by disease onset year, sex and age with non-HCW patients who had pulmonary TB and received treatment at Medical Center A (Fig 1).

#### Clinical Presentation and Disease Severity

Clinical presentations, disease severity, and microbiological results were similar between the cases and comparison groups (Table 3). None of the HCWs had MDR-TB, but the difference is not statistically significant (e.g., MDR-TB: 0/17 [0.0%] in the cases vs. 4/87 [4.6%] in the comparison group, P = 0.19) (Table 3).

**Table 3:** The characteristics of HCW TB patients vs. non-HCW TB patients.

	HCW (N = 30)		Non-HCW (N = 120)		P value
	n	%	n	%	
Male	7	23.3	28	23.3	N/A
Age	40.0±12.3		41.3±12.5		N/A
Married	18	60.0	63	52.5	0.43
Smoking	2	6.7	24	20.0	0.01
Alcohol consumption	4	13.3	17	14.2	0.89
Education: college or higher	26	86.7	40	44.4^a^	<0.001
Living in Taipei city or New Taipei county	24	80.0	99	82.5	0.75
**Underlying diseases/condition**					
DM	2	6.7	14	11.7	0.28
CKD	0	0.0	3	2.5	0.08
Steroid use	0	0.0	1	0.8	0.33
COPD	0	0.0	8	6.7	0.003
Autoimmune	1	3.3	0	0.0	0.33
Malignancy	2	6.7	17	14.2	0.25
Anti-TNF therapy	0	0.0	0	0.0	—
Silicosis	0	0.0	0	0.0	—
HIV positivity	0	0.0(0/9)	5	4.9(5/102)	0.35
No chronic disease	27	90.0	88	73.3	0.02
Past history of TB	0	0.0	9	7.5	0.01
**Detection scenarios**					
Symptomatic					
Fever	8	26.7	46	38.3	0.25
Cough	15	50.0	81	67.5	0.10
Night sweats	1	3.3	9	7.5	0.34
Malaise	2	6.7	12	10.0	0.49
Body weight loss	4	13.3	33	27.5	0.10
Haemoptysis	2	6.7	20	16.7	0.06
Dyspnoea	5	16.7	34	28.3	0.19
Non-symptomatic	6	20.0	14	11.7	0.22
Periodic physical check-up	3	10.0	9	7.5	0.68
Contact tracing	3	10.0	5	4.2	0.35
**Treatment delay (days)**	Median (IQR)		Median (IQR)		
Patient delay	12.5 (0–57)		29.8 (10.0–57.5)		0.11
Physician delay	0 (0–0)		0.6 (0.0–12.3)		0.007
Treatment delay^b^	20.0 (6.0–52.0)		42.6 (26.0–122.7)		0.02
Total delay	46.0 (31.0–104.0)		61.0 (28.0–110.0)		0.03
**Disease severity**					
Cavity lesion	10	33.3	9	7.5	0.01
Combined with extrapulmonary TB	3	10.0	17	14.2	0.52
**Culture result**					
Culture positive	17	56.7	87	72.5	0.15
Any resistance	1	5.9(1/17)	12	13.8(12/87)	0.37
Low-level INH resistance	1	5.9(1/17)	10	11.5(10/87)	0.45
High-level INH resistance	1	5.9(1/17)	4	4.6(4/87)	0.65
Low-level EMB resistance	0	0.0(0/17)	2	2.3(2/87)	0.33
High-level EMB resistance	0	0.0(0/17)	0	0.0(0/87)	—
RIF resistance	0	0.0(0/17)	6	5.7(6/87)	0.19
MDR TB	0	0.0(0/17)	4	4.6(4/87)	0.19
**Treatment**					
DOTS^c^	15	50.0	83	69.2	0.08
Hospitalised	11	36.7	44	36.7	1.00
Standard HERZ regimen	29^d^	96.7	99^e^	82.5	0.005
Initial HERZ	30	100	113	94.2	0.18
Regimen modification due to adverse effect or susceptibility report	1	3.3(1/30)	14	12.4(14/113)	0.15
Treatment duration (months) (mean ± SD)	7.7±0.4		8.6±0.9		0.64
**Side effects**					
Any	17	56.7	95	79.2	0.02
Blurred vision	1	3.3	22	18.3	0.008
Rash	3	10.0	25	20.8	0.15
Acute kidney injury	0	0.0	3	2.5	0.08
Malaise	3	10.0	9	7.5	0.65
GI upset	5	16.7	32	26.7	0.27
Itching	7	23.3	32	26.7	0.73
Hyperuricaemia	6	20.0	74	61.7	<0.001
Neutropenia	1	3.3	2	1.7	0.65
Hepatitis	4	13.3	6	5.0	0.18
**Compliance**					
No delay in follow up	26	86.7	102	85.0	0.76
Delay OPD follow up (days) (means with SD)	6.5±0.9		24.8±10.3		0.09
**Outcome**					
Complete TB therapy	30	100.0	112	93.3	0.003
TB-related mortality	0	0.0	7	5.8	0.008

DM: diabetes mellitus; CKD: chronic kidney disease; COPD: chronic occlusive pulmonary disease; TNF: tumour necrosis factor; INH: isoniazid; EMB: ethambutol; RIF: rifampicin; HERZ: treatment with INH, EMB, RIF and pyrazinamide; DOTS: directly observed treatment short-course; MDR TB: multidrug-resistant tuberculosis

^a^ 40/90, 30 non-HCW TB patients did not provide education data.

^b^ The time interval from the first diagnostic work-up (e.g., chest X-ray, sputum AFS/ *Mycobacterium* culture, or biopsy) to the initiation of anti-tuberculosis therapy. The causes of the delays included equivocal radiology/pathology findings, as well as a negative smear or culture results.

^c^ 15 HCWs did not participate in the DOTS programme.

^d^ One HCW received the standard HERZ regimen initially, but the regimen was subsequently modified due to adverse effects.

^e^ In 7 non-HCW patients, the initial treatment was not a standard HERZ treatment. Another 14 non-HCW patients received the standard HERZ regimen initially; however, the regimen was subsequently modified due to adverse effects (n = 12) or multi-drug resistance (MDR) (n = 3).

HCWs have a higher proportion of individuals who received a college or higher education compared with non-HCWs (26/30 [86.7%] vs. 40/90 [44.4%], P <0.001), and are more likely to have no underlying diseases (27/30 [90/0%] vs. 88/120 [73.3%], P = 0.02). In contrast, non-HCWs were more likely to be current/past smokers (24/120 [20.0%] vs. 2/30 [6.7%], P = 0.01) and were more likely to suffer chronic obstructive pulmonary disease (8/120 [6.7%] vs. 0/30 [0.0%], P = 0.003) or have past history of TB (9/120 [7.5%] vs. 0/30 [0.0%], P = 0.01). There is no difference in residential area between HCWs and non-HCWs (Table 3).

#### Timing of TB Diagnosis and Treatment

HCWs were significantly less likely to have a delay in diagnosis or a delay in treatment than non-HCWs [physician delay (mean, interquartile range) 0 (0–0) days vs. 0.6 (0.0–12.3) days, P = 0.007; treatment delay: 20.0 (6.0–52.0) days vs. 42.6 (26.0–122.7) days, P = 0.02; and total delay: 46.0 (31.0–104.0) days vs. 61.0 (28.0–110.0) days, P = 0.03].

With an annual CXR screening programme and contact tracing, more HCW-TB cases are being detected early in the asymptomatic stage compared with non-HCW TB cases (20% vs. 11.7%), although the difference did not reach statistical significance due to an insufficient sample size.

#### TB Treatment Outcome of HCWs

The majority of HCWs and non-HCWs patients received HERZ as the initial TB treatment regimen (30/30 [100.0%] in HCWs vs. 113/120 [94.2%] in non-HCWs, P = 0.35), but HCWs reported significantly fewer side effects during the treatment period than non-HCWs (17/30 [56.7%] vs. 95/120 [79.2%], P = 0.02) (Table 3).

None of the HCWs TB patients died. The 11 HCWs who were excluded for not receiving TB treatment at this hospital or having extrapulmonary TB also completed anti-TB treatment without mortality, according to national registry data. In contrast, eight non-HCW TB patients died during the course of anti-TB treatment. The mean age of the eight deceased patients was 48.5±8.0 years; 37.5% of them were male, and 50% (4/8) had culture-proven TB. One patient died on TB treatment day 390, although TB was not the main cause of death according to the death certificate. The other seven TB patients died within 30 days of TB diagnosis. The TB-related mortality rate was 0.0% (0/30) among the TB-HCWs and 5.8% among the non-HCW TB patients (7/120). The risk difference in TB-related mortality is −5.8% (95% CI: −10.1% ~ −1.5%, P = 0.008, Mantel-Haenszel test).

### Outcomes: Comparison with National TB Data

The standardized mortality rate of nationally registered TB patients in Taiwan was 1.08% (95% CI: 0.96% - 1.20%) (S2 Table). None of the HCWs died. The standardized mortality rate of HCWs TB patients was 0% (S2 Table).

## Discussion

Our results show that the incidence of active TB among the HCWs at the study hospital was significantly higher than that of the general population in Taiwan, after standardization by age, sex, and diagnosis year. However, the outcome of HCWs TB was significantly better than that of non-HCW patients treated in the same setting.

The increased incidence of active TB among HCWs compared with the age/sex-matched Taiwan general population in the present study is consistent with previous reports from other countries [16–27], except for those with an extremely low TB burden [28–30]. Occupational exposure is the most likely reason for this increase in the active TB incidence [1] although the epidemiological link with other TB cases is often difficult to establish in the absence of routine genotyping surveillance [7, 31]. Medical Center A identifies and treats a significant proportion of new TB cases in Taiwan (3.6% of all new TB cases in 2012) [32]. An atypical TB presentation compounded with an underlying complicated disease or health status poses a challenge to the rapid diagnosis of TB [33, 34]. Thus, the results of our study emphasise the importance of a TB control program for occupational safety, which is also equally important for patient safety.

The treatment outcomes of HCWs with active TB have seldom been reported. A small study conducted in British Columbia reported no significant difference in clinical features between 25 HCWs and 50 controls, with treatment completion rates of 84% in both of the study groups [4]. In contrast, two nationwide studies conducted in the USA [2, 3] reported high age-standardised and race- and sex-specific PMRs. From 1979–1990, the PMRs among individuals with a health service occupation were 350- (white male) to 24 (black male)-fold higher than that of the general population [2], and after one decade, they remained high among white males (1.78) and females (1.33) working in hospitals [3]. However, the PMR reflects a mixed effect from exposure risk, infection, and treatment prognosis. Our study showed that the studied HCWs indeed carried a higher risk of contracting TB infections; however, their TB-related mortality rate was significantly lower than that in the age/sex-matched general population.

To the best of our knowledge, the present study is the first cohort study to compare the treatment outcomes of HCWs with TB to those of age- and sex-matched non-HCW TB patients. In the present study, we further explored the reasons for the lower TB-related mortality rate in HCWs. The healthy worker effect was likely a factor because the HCWs had significantly fewer underlying chronic diseases. A high education level and associated socioeconomic status may also explain the lower mortality rate [35]. However, there were other factors that may explain the observed differences. For example, an important reason for the lower TB-related mortality rate in HCWs is the more rapid diagnoses and treatments. The HCWs in our study suffered from significantly fewer physician and treatment delays. Delays in TB diagnosis and/or treatment have been shown to be associated with an increase in clinical severity and an increased risk of subsequent COPD [36, 37]. In one study, implementation of a hospital-wide program that reduced the health provider delay led to a decrease in mortality among TB patients [38]. Our findings add to such evidence, indicating that prompt recognition and treatment are crucial for improving TB outcome.

We observed that nearly all of the HCW-TB patients (29/30, 96.7%) completed the standard HERZ treatment. In a study conducted in Finland that included 629 TB patients, completion of a standard HRZ regimen was significantly associated with a decreased risk of unfavourable outcomes, including death, treatment failure or default [39]. In our study, the most common reasons for failure to complete the standard regimen among non-HCW TB patients were adverse drug effects (n = 12), followed by a susceptibility test that revealed multi-drug resistance (n = 3), both of which necessitate a switch to an alternative regimen. It is interesting to note that the rates of hyperuricemia is very high in the non-HCWs of our study. In Japan, hyperuricemia (serum uric acid ≥ 8 mg/dL) has been reported to occur in 84.5% of TB patients during pyrazinamide (PZA) therapy [40]. In our study, hyperuricemia (defined as serum uric acid ≥ 7.5 mg/dL) occurred in 61.7% of the non-HCW TB patients during TB therapy. It appears that Asian patients are prone to PZA-induced hyperuricemia. However, more studies are needed for a definite conclusion on this issue. The lower incidence of drug side effects in HCWs of our study might be explained by their generally healthy status. The resulted higher rate of completing standard HERZ regimen may also contributed to a better TB treatment outcome in HCWs.

Another possible factor for the lower TB-related mortality rate in our study was the lack of MDR-TB cases among the studied HCWs. Indeed, MDR or extensively drug-resistant TB is a significant risk factor for TB mortality [41, 42]. Since 2007, all MDR-TB patients in Taiwan were referred to five specialized MDR-TB Centers that provide DOTS-Plus programs [43]. This policy may have reduced the nosocomial MDR-TB transmission to HCWs in general hospitals, and contributed to the low observed TB-related mortality rate in our study. The MDR-TB prevalence among new TB patients in Taiwan was 1.1% in 2011 [32].

Our study has several limitations. First, the study was conducted at a medical center, and therefore, our results may not be applicable to primary care settings. Nonetheless, under the national health insurance system in Taiwan, patients usually seek primary care at medical centers. The residential address distributions were similar between the HCW-TB and non-HCW TB groups in our study, which strengthened the comparability between the two groups. Another important limitation was the low MDR-TB case percentage at the study hospital; therefore, our conclusions may not be applicable to settings with MDR-TB outbreaks. Finally, because of the low HIV prevalence in Taiwan (0.16% of the adult population) [44], none of our HCW-TB subjects were HIV-positive, which contributed to their good outcomes. Our observations thus may not be applied to countries with high HIV burdens.

In conclusion, our study shows that compared with age- and sex-matched Taiwan general population, Taiwanese HCWs have a significantly higher risk of active TB. To mitigate this occupational hazard, more efforts need to be towards preventing the nosocomial transmission of TB to HCWs. Compared with age- and sex-matched non-HCW TB patients, HCWs have a significantly better TB treatment outcome. Healthy worker effect, more rapid diagnosis, and less delay in treatment, contributed to a lower TB mortality in HCWs.

## Supporting Information

S1 Dataset (XLSX)

S1 Table Standardized TB incidence ratio. (DOCX)

S2 Table Standardized TB mortality rate. (DOCX)
