# Supplementary material for: Tuberculosis in Healthcare Workers: A Matched Cohort Study in Taiwan
Source: PLoS One. 2015 Dec 17;10(12):e0145047. doi: 10.1371/journal.pone.0145047 (PMC4683009; doi:10.1371/journal.pone.0145047)
Supplement: S1 Table — (DOCX) [file pone.0145047.s002.docx]

Appendix table S1. Expected and observed active TB cases among HCWs compared with the general population in Taiwan

| Male | | | | | Female | | | | |
| --- | --- | --- | --- | --- | --- | --- | --- | --- | --- |
| Age categories | Year | TB incidence among the general population (I) | Observed population (P) | Expected cases (I*P) | Age categories | Year | TB incidence among the general population (I) | Observed population (P) | Expected cases (I*P) |
| 15-24 | 2006 | 0.0002 | 0 | 0 | 15-24 | 2006 | 0.0003 | 0 | 0 |
| 15-24 | 2007 | 0.0002 | 0 | 0 | 15-24 | 2007 | 0.0002 | 0 | 0 |
| 15-24 | 2008 | 0.0002 | 0 | 0 | 15-24 | 2008 | 0.0002 | 0 | 0 |
| 15-24 | 2009 | 0.0002 | 0 | 0 | 15-24 | 2009 | 0.0002 | 0 | 0 |
| 15-24 | 2010 | 0.0002 | 0 | 0 | 15-24 | 2010 | 0.0002 | 0 | 0 |
| 15-24 | 2011 | 0.0002 | 429 | 0.08 | 15-24 | 2011 | 0.0002 | 17 | 0 |
| 15-24 | 2012 | 0.0001 | 0 | 0 | 15-24 | 2012 | 0.0002 | 0 | 0 |
| 25-34 | 2006 | 0.0003 | 454 | 0.12 | 25-34 | 2006 | 0.0003 | 17 | 0.01 |
| 25-34 | 2007 | 0.0003 | 650 | 0.16 | 25-34 | 2007 | 0.0003 | 50 | 0.01 |
| 25-34 | 2008 | 0.0002 | 1111 | 0.28 | 25-34 | 2008 | 0.0003 | 142 | 0.04 |
| 25-34 | 2009 | 0.0002 | 1319 | 0.29 | 25-34 | 2009 | 0.0002 | 231 | 0.05 |
| 25-34 | 2010 | 0.0002 | 1528 | 0.35 | 25-34 | 2010 | 0.0002 | 318 | 0.07 |
| 25-34 | 2011 | 0.0002 | 2071 | 0.43 | 25-34 | 2011 | 0.0002 | 619 | 0.13 |
| 25-34 | 2012 | 0.0002 | 2151 | 0.39 | 25-34 | 2012 | 0.0002 | 537 | 0.11 |
| 35-44 | 2006 | 0.0003 | 1556 | 0.39 | 35-44 | 2006 | 0.0005 | 520 | 0.26 |
| 35-44 | 2007 | 0.0003 | 1496 | 0.38 | 35-44 | 2007 | 0.0004 | 524 | 0.23 |
| 35-44 | 2008 | 0.0002 | 1529 | 0.36 | 35-44 | 2008 | 0.0005 | 509 | 0.23 |
| 35-44 | 2009 | 0.0002 | 1479 | 0.31 | 35-44 | 2009 | 0.0004 | 471 | 0.18 |
| 35-44 | 2010 | 0.0002 | 1417 | 0.29 | 35-44 | 2010 | 0.0004 | 427 | 0.16 |
| 35-44 | 2011 | 0.0002 | 1096 | 0.22 | 35-44 | 2011 | 0.0003 | 292 | 0.1 |
| 35-44 | 2012 | 0.0002 | 1345 | 0.25 | 35-44 | 2012 | 0.0003 | 370 | 0.12 |
| 45-54 | 2006 | 0.0003 | 858 | 0.29 | 45-54 | 2006 | 0.0009 | 297 | 0.26 |
| 45-54 | 2007 | 0.0003 | 846 | 0.27 | 45-54 | 2007 | 0.0007 | 273 | 0.2 |
| 45-54 | 2008 | 0.0003 | 852 | 0.25 | 45-54 | 2008 | 0.0007 | 263 | 0.18 |
| 45-54 | 2009 | 0.0003 | 853 | 0.21 | 45-54 | 2009 | 0.0007 | 268 | 0.18 |
| 45-54 | 2010 | 0.0002 | 848 | 0.2 | 45-54 | 2010 | 0.0007 | 264 | 0.18 |
| 45-54 | 2011 | 0.0002 | 562 | 0.13 | 45-54 | 2011 | 0.0006 | 230 | 0.15 |
| 45-54 | 2012 | 0.0002 | 834 | 0.19 | 45-54 | 2012 | 0.0006 | 260 | 0.16 |
| 55-64 | 2006 | 0.0006 | 302 | 0.17 | 55-64 | 2006 | 0.0015 | 161 | 0.24 |
| 55-64 | 2007 | 0.0004 | 283 | 0.13 | 55-64 | 2007 | 0.0014 | 150 | 0.21 |
| 55-64 | 2008 | 0.0004 | 254 | 0.11 | 55-64 | 2008 | 0.0012 | 148 | 0.18 |
| 55-64 | 2009 | 0.0004 | 237 | 0.1 | 55-64 | 2009 | 0.0011 | 139 | 0.15 |
| 55-64 | 2010 | 0.0004 | 214 | 0.09 | 55-64 | 2010 | 0.0011 | 131 | 0.15 |
| 55-64 | 2011 | 0.0004 | 79 | 0.03 | 55-64 | 2011 | 0.0011 | 51 | 0.05 |
| 55-64 | 2012 | 0.0004 | 181 | 0.07 | 55-64 | 2012 | 0.0011 | 114 | 0.12 |
| 65+ | 2006 | 0.0019 | 34 | 0.07 | 65+ | 2006 | 0.005 | 26 | 0.13 |
| 65+ | 2007 | 0.0017 | 28 | 0.05 | 65+ | 2007 | 0.0047 | 25 | 0.12 |
| 65+ | 2008 | 0.0017 | 21 | 0.04 | 65+ | 2008 | 0.0046 | 22 | 0.1 |
| 65+ | 2009 | 0.0015 | 15 | 0.02 | 65+ | 2009 | 0.0043 | 19 | 0.08 |
| 65+ | 2010 | 0.0015 | 10 | 0.02 | 65+ | 2010 | 0.0042 | 15 | 0.06 |
| 65+ | 2011 | 0.0014 | 1 | 0 | 65+ | 2011 | 0.004 | 2 | 0.01 |
| 65+ | 2012 | 0.0013 | 4 | 0.01 | 65+ | 2012 | 0.0038 | 11 | 0.04 |

Observed cases: 22

Expected cases: 11.41

Standard incidence ratio: 1.9 (95% CI: 1.2-2.9)
