# Supplementary material for: Tuberculosis in Healthcare Workers: A Matched Cohort Study in Taiwan
Source: PLoS One. 2015 Dec 17;10(12):e0145047. doi: 10.1371/journal.pone.0145047 (PMC4683009; doi:10.1371/journal.pone.0145047)
Supplement: S2 Table — (DOCX) [file pone.0145047.s003.docx]

Appendix table S2-1. Standardised TB mortality rate of nationally registered TB cases based on the population structure of HCWs at medical center A

| Male | | | | | | | Female | | | | | | |
| --- | --- | --- | --- | --- | --- | --- | --- | --- | --- | --- | --- | --- | --- |
| Age | Year | Registered TB cases | Mortality cases | Stratum mortality rate (R) | Standard population proportion (P) | R*P | Age | Year | Registered TB cases | Mortality cases | Stratum mortality rate (R) | Standard population proportion (P) | R*P |
| 15-24 | 2004 | 498 | 1 | 0.002 | 0 | 0 | 15-24 | 2004 | 451 | 1 | 0.0022 | 0 | 0 |
| 15-24 | 2005 | 498 | 1 | 0.002 | 0 | 0 | 15-24 | 2005 | 451 | 1 | 0.0022 | 0 | 0 |
| 15-24 | 2006 | 464 | 0 | 0 | 0 | 0 | 15-24 | 2006 | 388 | 2 | 0.0052 | 0 | 0 |
| 15-24 | 2007 | 409 | 1 | 0.0024 | 0 | 0 | 15-24 | 2007 | 367 | 2 | 0.0054 | 0 | 0 |
| 15-24 | 2008 | 418 | 3 | 0.0072 | 0 | 0 | 15-24 | 2008 | 335 | 0 | 0 | 0 | 0 |
| 15-24 | 2009 | 396 | 1 | 0.0025 | 0 | 0 | 15-24 | 2009 | 286 | 2 | 0.007 | 0 | 0 |
| 15-24 | 2010 | 345 | 0 | 0 | 0 | 0 | 15-24 | 2010 | 287 | 2 | 0.007 | 0 | 0 |
| 15-24 | 2011 | 350 | 0 | 0 | 0 | 0 | 15-24 | 2011 | 298 | 1 | 0.0034 | 0 | 0 |
| 15-24 | 2012 | 329 | 0 | 0 | 0 | 0 | 15-24 | 2012 | 227 | 0 | 0 | 0 | 0 |
| 25-34 | 2004 | 682 | 6 | 0.0088 | 0.01 | 0.0001 | 25-34 | 2004 | 600 | 4 | 0.0067 | 0.041 | 0.0003 |
| 25-34 | 2005 | 682 | 6 | 0.0088 | 0.01 | 0.0001 | 25-34 | 2005 | 600 | 4 | 0.0067 | 0.041 | 0.0003 |
| 25-34 | 2006 | 576 | 5 | 0.0087 | 0.01 | 0.0001 | 25-34 | 2006 | 498 | 1 | 0.002 | 0.041 | 0.0001 |
| 25-34 | 2007 | 530 | 4 | 0.0075 | 0.01 | 0.0001 | 25-34 | 2007 | 488 | 2 | 0.0041 | 0.041 | 0.0002 |
| 25-34 | 2008 | 560 | 5 | 0.0089 | 0.01 | 0.0001 | 25-34 | 2008 | 483 | 0 | 0 | 0.041 | 0 |
| 25-34 | 2009 | 469 | 3 | 0.0064 | 0.01 | 0.0001 | 25-34 | 2009 | 423 | 4 | 0.0095 | 0.041 | 0.0004 |
| 25-34 | 2010 | 456 | 6 | 0.0132 | 0.01 | 0.0001 | 25-34 | 2010 | 452 | 2 | 0.0044 | 0.041 | 0.0002 |
| 25-34 | 2011 | 414 | 1 | 0.0024 | 0.01 | 0 | 25-34 | 2011 | 355 | 1 | 0.0028 | 0.041 | 0.0001 |
| 25-34 | 2012 | 404 | 4 | 0.0099 | 0.01 | 0.0001 | 25-34 | 2012 | 335 | 3 | 0.009 | 0.041 | 0.0004 |
| 35-44 | 2004 | 1045 | 24 | 0.023 | 0.007 | 0.0002 | 35-44 | 2004 | 490 | 2 | 0.0041 | 0.026 | 0.0001 |
| 35-44 | 2005 | 1045 | 24 | 0.023 | 0.007 | 0.0002 | 35-44 | 2005 | 490 | 2 | 0.0041 | 0.026 | 0.0001 |
| 35-44 | 2006 | 906 | 23 | 0.0254 | 0.007 | 0.0002 | 35-44 | 2006 | 467 | 3 | 0.0064 | 0.026 | 0.0002 |
| 35-44 | 2007 | 833 | 23 | 0.0276 | 0.007 | 0.0002 | 35-44 | 2007 | 477 | 3 | 0.0063 | 0.026 | 0.0002 |
| 35-44 | 2008 | 849 | 13 | 0.0153 | 0.007 | 0.0001 | 35-44 | 2008 | 430 | 4 | 0.0093 | 0.026 | 0.0002 |
| 35-44 | 2009 | 694 | 14 | 0.0202 | 0.007 | 0.0001 | 35-44 | 2009 | 390 | 6 | 0.0154 | 0.026 | 0.0004 |
| 35-44 | 2010 | 664 | 7 | 0.0105 | 0.007 | 0.0001 | 35-44 | 2010 | 382 | 3 | 0.0079 | 0.026 | 0.0002 |
| 35-44 | 2011 | 630 | 12 | 0.019 | 0.007 | 0.0001 | 35-44 | 2011 | 403 | 3 | 0.0074 | 0.026 | 0.0002 |
| 35-44 | 2012 | 620 | 12 | 0.0194 | 0.007 | 0.0001 | 35-44 | 2012 | 352 | 3 | 0.0085 | 0.026 | 0.0002 |
| 45-54 | 2004 | 1546 | 52 | 0.0336 | 0.005 | 0.0002 | 45-54 | 2004 | 604 | 11 | 0.0182 | 0.016 | 0.0003 |
| 45-54 | 2005 | 1546 | 52 | 0.0336 | 0.005 | 0.0002 | 45-54 | 2005 | 604 | 11 | 0.0182 | 0.016 | 0.0003 |
| 45-54 | 2006 | 1439 | 45 | 0.0313 | 0.005 | 0.0002 | 45-54 | 2006 | 551 | 6 | 0.0109 | 0.016 | 0.0002 |
| 45-54 | 2007 | 1299 | 35 | 0.0269 | 0.005 | 0.0001 | 45-54 | 2007 | 574 | 6 | 0.0105 | 0.016 | 0.0002 |
| 45-54 | 2008 | 1249 | 40 | 0.032 | 0.005 | 0.0002 | 45-54 | 2008 | 533 | 4 | 0.0075 | 0.016 | 0.0001 |
| 45-54 | 2009 | 1214 | 32 | 0.0264 | 0.005 | 0.0001 | 45-54 | 2009 | 461 | 12 | 0.026 | 0.016 | 0.0004 |
| 45-54 | 2010 | 1217 | 31 | 0.0255 | 0.005 | 0.0001 | 45-54 | 2010 | 428 | 7 | 0.0164 | 0.016 | 0.0003 |
| 45-54 | 2011 | 1169 | 25 | 0.0214 | 0.005 | 0.0001 | 45-54 | 2011 | 394 | 7 | 0.0178 | 0.016 | 0.0003 |
| 45-54 | 2012 | 1159 | 36 | 0.0311 | 0.005 | 0.0002 | 45-54 | 2012 | 415 | 4 | 0.0096 | 0.016 | 0.0002 |
| 55-64 | 2004 | 1500 | 67 | 0.0447 | 0.002 | 0.0001 | 55-64 | 2004 | 530 | 13 | 0.0245 | 0.003 | 0.0001 |
| 55-64 | 2005 | 1500 | 67 | 0.0447 | 0.002 | 0.0001 | 55-64 | 2005 | 530 | 13 | 0.0245 | 0.003 | 0.0001 |
| 55-64 | 2006 | 1380 | 54 | 0.0391 | 0.002 | 0.0001 | 55-64 | 2006 | 566 | 16 | 0.0283 | 0.003 | 0.0001 |
| 55-64 | 2007 | 1431 | 61 | 0.0426 | 0.002 | 0.0001 | 55-64 | 2007 | 480 | 7 | 0.0146 | 0.003 | 0.0001 |
| 55-64 | 2008 | 1347 | 41 | 0.0304 | 0.002 | 0.0001 | 55-64 | 2008 | 498 | 18 | 0.0361 | 0.003 | 0.0001 |
| 55-64 | 2009 | 1301 | 51 | 0.0392 | 0.002 | 0.0001 | 55-64 | 2009 | 524 | 11 | 0.021 | 0.003 | 0.0001 |
| 55-64 | 2010 | 1383 | 42 | 0.0304 | 0.002 | 0.0001 | 55-64 | 2010 | 542 | 8 | 0.0148 | 0.003 | 0.0001 |
| 55-64 | 2011 | 1389 | 33 | 0.0238 | 0.002 | 0.0001 | 55-64 | 2011 | 498 | 9 | 0.0181 | 0.003 | 0.0001 |
| 55-64 | 2012 | 1474 | 36 | 0.0244 | 0.002 | 0.0001 | 55-64 | 2012 | 534 | 8 | 0.015 | 0.003 | 0.0001 |
| 65+ | 2004 | 6077 | 591 | 0.0973 | 0 | 0 | 65+ | 2004 | 2331 | 197 | 0.0845 | 0 | 0 |
| 65+ | 2005 | 6077 | 591 | 0.0973 | 0 | 0 | 65+ | 2005 | 2331 | 197 | 0.0845 | 0 | 0 |
| 65+ | 2006 | 5784 | 480 | 0.083 | 0 | 0 | 65+ | 2006 | 2245 | 197 | 0.0878 | 0 | 0 |
| 65+ | 2007 | 5426 | 494 | 0.091 | 0 | 0 | 65+ | 2007 | 2051 | 143 | 0.0697 | 0 | 0 |
| 65+ | 2008 | 5351 | 475 | 0.0888 | 0 | 0 | 65+ | 2008 | 2100 | 157 | 0.0748 | 0 | 0 |
| 65+ | 2009 | 5135 | 463 | 0.0902 | 0 | 0 | 65+ | 2009 | 1943 | 146 | 0.0751 | 0 | 0 |
| 65+ | 2010 | 5027 | 390 | 0.0776 | 0 | 0 | 65+ | 2010 | 1973 | 156 | 0.0791 | 0 | 0 |
| 65+ | 2011 | 4788 | 408 | 0.0852 | 0 | 0 | 65+ | 2011 | 218 | 138 | 0.633 | 0 | 0 |
| 65+ | 2012 | 4637 | 391 | 0.0843 | 0 | 0 | 65+ | 2012 | 1787 | 128 | 0.0716 | 0 | 0 |

Crude mortality rate: 5.53%

Adjusted mortality rate: 1.08% (95% CI: 0.96-1.20%)

Appendix table S2-2. Standardised TB mortality rate of HCW-TB cases based on the population structure of HCWs at medical center A

| Age | Year | HCW TB cases | Mortality cases | Stratum mortality rate (R) | Standard population proportion (P) | R*P | Age | Year | HCW TB cases | Mortality cases | Stratum mortality rate (R) | Standard population proportion (P) | R*P |
| --- | --- | --- | --- | --- | --- | --- | --- | --- | --- | --- | --- | --- | --- |
| 15-24 | 2004 | 0 | 0 | . | 0 | . | 15-24 | 2004 | 1 | 0 | 0 | 0 | 0 |
| 15-24 | 2005 | 0 | 0 | . | 0 | . | 15-24 | 2005 | 1 | 0 | 0 | 0 | 0 |
| 15-24 | 2006 | 1 | 0 | 0 | 0 | 0 | 15-24 | 2006 | 0 | 0 | . | 0 | . |
| 15-24 | 2007 | 0 | 0 | . | 0 | . | 15-24 | 2007 | 0 | 0 | . | 0 | . |
| 15-24 | 2008 | 0 | 0 | . | 0 | . | 15-24 | 2008 | 0 | 0 | . | 0 | . |
| 15-24 | 2009 | 0 | 0 | . | 0 | . | 15-24 | 2009 | 0 | 0 | . | 0 | . |
| 15-24 | 2010 | 0 | 0 | . | 0 | . | 15-24 | 2010 | 0 | 0 | . | 0 | . |
| 15-24 | 2011 | 0 | 0 | . | 0 | . | 15-24 | 2011 | 0 | 0 | . | 0 | . |
| 15-24 | 2012 | 0 | 0 | . | 0 | . | 15-24 | 2012 | 0 | 0 | . | 0 | . |
| 25-34 | 2004 | 0 | 0 | . | 0.041 | . | 25-34 | 2004 | 0 | 0 | . | 0.01 | . |
| 25-34 | 2005 | 4 | 0 | 0 | 0.041 | 0 | 25-34 | 2005 | 0 | 0 | . | 0.01 | . |
| 25-34 | 2006 | 1 | 0 | 0 | 0.041 | 0 | 25-34 | 2006 | 0 | 0 | . | 0.01 | . |
| 25-34 | 2007 | 0 | 0 | . | 0.041 | . | 25-34 | 2007 | 0 | 0 | . | 0.01 | . |
| 25-34 | 2008 | 2 | 0 | 0 | 0.041 | 0 | 25-34 | 2008 | 0 | 0 | . | 0.01 | . |
| 25-34 | 2009 | 2 | 0 | 0 | 0.041 | 0 | 25-34 | 2009 | 1 | 0 | 0 | 0.01 | 0 |
| 25-34 | 2010 | 1 | 0 | 0 | 0.041 | 0 | 25-34 | 2010 | 0 | 0 | . | 0.01 | . |
| 25-34 | 2011 | 1 | 0 | 0 | 0.041 | 0 | 25-34 | 2011 | 1 | 0 | 0 | 0.01 | 0 |
| 25-34 | 2012 | 2 | 0 | 0 | 0.041 | 0 | 25-34 | 2012 | 0 | 0 | . | 0.01 | . |
| 35-44 | 2004 | 1 | 0 | 0 | 0.026 | 0 | 35-44 | 2004 | 0 | 0 | . | 0.007 | . |
| 35-44 | 2005 | 0 | 0 | . | 0.026 | . | 35-44 | 2005 | 2 | 0 | 0 | 0.007 | 0 |
| 35-44 | 2006 | 0 | 0 | . | 0.026 | . | 35-44 | 2006 | 0 | 0 | . | 0.007 | . |
| 35-44 | 2007 | 1 | 0 | 0 | 0.026 | 0 | 35-44 | 2007 | 0 | 0 | . | 0.007 | . |
| 35-44 | 2008 | 1 | 0 | 0 | 0.026 | 0 | 35-44 | 2008 | 0 | 0 | . | 0.007 | . |
| 35-44 | 2009 | 1 | 0 | 0 | 0.026 | 0 | 35-44 | 2009 | 0 | 0 | . | 0.007 | . |
| 35-44 | 2010 | 1 | 0 | 0 | 0.026 | 0 | 35-44 | 2010 | 0 | 0 | . | 0.007 | . |
| 35-44 | 2011 | 0 | 0 | . | 0.026 | . | 35-44 | 2011 | 0 | 0 | . | 0.007 | . |
| 35-44 | 2012 | 0 | 0 | . | 0.026 | . | 35-44 | 2012 | 0 | 0 | . | 0.007 | . |
| 45-54 | 2004 | 2 | 0 | 0 | 0.016 | 0 | 45-54 | 2004 | 0 | 0 | . | 0.005 | . |
| 45-54 | 2005 | 0 | 0 | . | 0.016 | . | 45-54 | 2005 | 0 | 0 | . | 0.005 | . |
| 45-54 | 2006 | 1 | 0 | 0 | 0.016 | 0 | 45-54 | 2006 | 2 | 0 | 0 | 0.005 | 0 |
| 45-54 | 2007 | 0 | 0 | . | 0.016 | . | 45-54 | 2007 | 0 | 0 | . | 0.005 | . |
| 45-54 | 2008 | 0 | 0 | . | 0.016 | . | 45-54 | 2008 | 0 | 0 | . | 0.005 | . |
| 45-54 | 2009 | 0 | 0 | . | 0.016 | . | 45-54 | 2009 | 2 | 0 | 0 | 0.005 | 0 |
| 45-54 | 2010 | 2 | 0 | 0 | 0.016 | 0 | 45-54 | 2010 | 0 | 0 | . | 0.005 | . |
| 45-54 | 2011 | 0 | 0 | . | 0.016 | . | 45-54 | 2011 | 1 | 0 | 0 | 0.005 | 0 |
| 45-54 | 2012 | 1 | 0 | 0 | 0.016 | 0 | 45-54 | 2012 | 0 | 0 | . | 0.005 | . |
| 55-64 | 2004 | 1 | 0 | 0 | 0.003 | 0 | 55-64 | 2004 | 0 | 0 | . | 0.002 | . |
| 55-64 | 2005 | 0 | 0 | . | 0.003 | . | 55-64 | 2005 | 0 | 0 | . | 0.002 | . |
| 55-64 | 2006 | 0 | 0 | . | 0.003 | . | 55-64 | 2006 | 1 | 0 | 0 | 0.002 | 0 |
| 55-64 | 2007 | 1 | 0 | 0 | 0.003 | 0 | 55-64 | 2007 | 0 | 0 | . | 0.002 | . |
| 55-64 | 2008 | 0 | 0 | . | 0.003 | . | 55-64 | 2008 | 0 | 0 | . | 0.002 | . |
| 55-64 | 2009 | 0 | 0 | . | 0.003 | . | 55-64 | 2009 | 0 | 0 | . | 0.002 | . |
| 55-64 | 2010 | 0 | 0 | . | 0.003 | . | 55-64 | 2010 | 0 | 0 | . | 0.002 | . |
| 55-64 | 2011 | 0 | 0 | . | 0.003 | . | 55-64 | 2011 | 0 | 0 | . | 0.002 | . |
| 55-64 | 2012 | 1 | 0 | 0 | 0.003 | 0 | 55-64 | 2012 | 0 | 0 | . | 0.002 | . |
| 65+ | 2004 | 0 | 0 | . | 0 | . | 65+ | 2004 | 0 | 0 | . | 0 | . |
| 65+ | 2005 | 0 | 0 | . | 0 | . | 65+ | 2005 | 0 | 0 | . | 0 | . |
| 65+ | 2006 | 0 | 0 | . | 0 | . | 65+ | 2006 | 0 | 0 | . | 0 | . |
| 65+ | 2007 | 0 | 0 | . | 0 | . | 65+ | 2007 | 0 | 0 | . | 0 | . |
| 65+ | 2008 | 0 | 0 | . | 0 | . | 65+ | 2008 | 0 | 0 | . | 0 | . |
| 65+ | 2009 | 0 | 0 | . | 0 | . | 65+ | 2009 | 0 | 0 | . | 0 | . |
| 65+ | 2010 | 1 | 0 | 0 | 0 | 0 | 65+ | 2010 | 0 | 0 | . | 0 | . |
| 65+ | 2011 | 0 | 0 | . | 0 | . | 65+ | 2011 | 0 | 0 | . | 0 | . |
| 65+ | 2012 | 0 | 0 | . | 0 | . | 65+ | 2012 | 0 | 0 | . | 0 | . |

Crude mortality rate: 0.00%

Adjusted mortality rate: 0.00%
